# Targets of miR-200c mediate suppression of cell motility and anoikis resistance

**DOI:** 10.1186/bcr2867

**Published:** 2011-04-18

**Authors:** Erin N Howe, Dawn R Cochrane, Jennifer K Richer

**Affiliations:** 1 Program in Cancer Biology, Department of Pathology, University of Colorado, Anschutz Medical Campus, Mail Stop 8104, P.O. Box 6511, Aurora, CO, USA; 2 Department of Pathology, University of Colorado, Anschutz Medical Campus, Mail Stop 8104, P.O. Box 6511, Aurora, CO, USA

## Abstract

**Introduction:**

miR-200c and other members of the miR-200 family promote epithelial identity by directly targeting ZEB1 and ZEB2, which repress E-cadherin and other genes involved in polarity. Loss of miR-200c is often observed in carcinoma cells that have undergone epithelial to mesenchymal transition (EMT). Restoration of miR-200c to such cells leads to a reduction in stem cell-like characteristics, reduced migration and invasion, and increased sensitivity to taxanes. Here we investigate the functional role of novel targets of miR-200c in the aggressive behavior of breast and endometrial cancer cells.

**Methods:**

Putative target genes of miR-200c identified by microarray profiling were validated as direct targets using dual luciferase reporter assays. Following restoration of miR-200c to triple negative breast cancer and type 2 endometrial cancer cell lines that had undergone EMT, levels of endogenous target mRNA and respective protein products were measured. Migration and sensitivity to anoikis were determined using wound healing assays or cell-death ELISAs and viability assays respectively.

**Results:**

We found that restoration of miR-200c suppresses anoikis resistance, a novel function for this influential miRNA. We identified novel targets of miR-200c, including genes encoding fibronectin 1 (FN1), moesin (MSN), neurotrophic tyrosine receptor kinase type 2 (NTRK2 or TrkB), leptin receptor (LEPR), and Rho GTPase activating protein 19 (ARHGAP19). These targets all encode proteins normally expressed in cells of mesenchymal or neuronal origin; however, in carcinoma cells that lack miR-200c they become aberrantly expressed and contribute to the EMT phenotype and aggressive behavior. We showed that these targets are inhibited upon restoration of miR-200c to aggressive breast and endometrial cancer cells. We demonstrated that inhibition of MSN and/or FN1 is sufficient to mediate the ability of miR-200c to suppress cell migration. Lastly, we showed that targeting of *TrkB *mediates the ability of miR-200c to restore anoikis sensitivity.

**Conclusions:**

miR-200c maintains the epithelial phenotype not only by targeting ZEB1/2, which usually facilitates restoration of E-cadherin expression, but also by actively repressing a program of mesenchymal and neuronal genes involved in cell motility and anoikis resistance.

## Introduction

Epithelial to mesenchymal transition (EMT) occurs during development as it is required for formation of the neural crest and palate, among other processes [1,2]. In cancer it is a pathological event associated with tumor progression and is thought to influence certain steps in the metastatic cascade, thereby contributing to the metastatic potential of carcinomas. Specifically, EMT likely contributes to the ability of carcinoma cells to invade through basement membrane and stroma and to intravasate into blood and lymph vessels [3-5]. The process of EMT is regulated by several transcription factors, including Twist, SNAIL, SLUG, ZEB1 (zinc finger E-box binding homeobox 1) and the closely related SIP1 (ZEB2), as reviewed in [6], which are transcriptional repressors of *E-cadherin*.

The miR-200 family of miRNAs, which includes miR-200c and miR-141 on chromosome 12 and miR-200a/b and miR-429 on chromosome 1, directly targets ZEB1 and ZEB2 [7-10]. Restoring miR-200c to aggressive breast, endometrial and ovarian cancer cells substantially decreases migration and invasion [9-13]. Since ZEB1 represses E-cadherin [14] and other genes involved in polarity [15], the reduction in migratory and invasive capacity observed when miR-200c is restored to cancer cells is widely thought to be due to the ability of miR-200c to target and repress ZEB1/2 which, in most cases, allows E-cadherin to be re-expressed. However, even in cell lines in which E-cadherin is not restored, miR-200c still dramatically reduces migration and invasion [11], implying that additional miR-200c targets can facilitate its ability to suppress cell motility.

We identify and confirm novel direct targets of miR-200c, including the genes encoding fibronectin 1 (FN1), moesin (MSN), neurotrophic tyrosine receptor kinase type 2 (NTRK2 or TrkB), leptin receptor (LEPR), and Rho GTPase activating protein 19 (ARHGAP19). These targets are all genes usually expressed in cells of mesenchymal or neuronal origin. However, in carcinoma cells that lack miR-200c, repression of these genes is compromised and they are allowed to be translated and contribute to an EMT phenotype and aggressive behavior. Here we show that *MSN *and *FN1 *are direct targets of miR-200c that contribute to the ability of miR-200c to suppress migration. We also identify a completely novel role for miR-200c - the ability to reverse anoikis resistance and we further pinpoint *TrkB *as the direct target that mediates this effect. Anoikis resistance is an important, yet understudied, step in the metastatic cascade.

## Materials and methods

### Cell culture

Hec50 cells were cultured in DMEM with 10% fetal bovine serum (FBS) and 2 mM L-glutamine. AN3CA cells and Ishikawa cells were grown in MEM with 5% FBS, nonessential amino acids (NEAA), penicillin, streptomycin and 1 nM insulin. MCF-7 cells were grown in DMEM with 10% FBS, and 2 mM L-glutamine. MDA-MB-231 cells were grown in MEM with 5% FBS, HEPES, NEAA, 2 mM L-glutamine, penicillin, streptomycin, and insulin. BT549 cells were grown in RPMI supplemented with 10% FBS and insulin. All cells were grown in a 37°C incubator with 5% CO_2_. Cell line identities were authenticated by isolating genomic DNA using ZR genomic DNAII kit (Zymo Research, Irvine, CA, USA) and DNA profiling multiplex PCR was performed using the Identifiler Kit (Applied Biosystems, Carlsbad, CA, USA) in the UC Cancer Center DNA Sequencing and Analysis Core.

### Transfection

miR-200c (miRNA mimic) or scrambled negative control (Ambion, Austin, TX, USA) at a concentration of 50 nM were incubated with Lipofectamine 2000 (Invitrogen, Carlsbad, CA, USA) in culture medium per the manufacturer's instructions before addition to cells. Cells were incubated at 37°C for 24 hrs before replacement of medium.

### DNA and shRNA constructs

pEGP-MSN (created by Stephen Shaw, National Institutes of Health, purchased from Addgene plasmid 20671, Cambridge, MA, USA) [16]. *FN1 *was subcloned from pCR-XL-TOPO-FN1 (Open Biosystems, Catalog number MHS4426-99240322, Huntsville, AL, USA) into pcDNA3.1 (Invitrogen). *TrkB *was subcloned from pBabe-TrkB (a gift from D. Peeper) into pcDNA3.1.

### Microarray analysis

Expression profiling was performed on Hec50 cells transfected as described above and statistical analysis was performed as described previously [12]. Array data have been provided to GEO, accession GSE25332. The heatmap was generated using GeneSpring GX 11 (Agilent, Santa Clara, CA, USA) and shows genes that are statistically significantly down-regulated by at least 1.5-fold in the miR-200c treated samples as compared to either the mock or scrambled control or both, and are predicted to be direct targets of miR-200c. Target site predictions were taken from TargetScan [17], http://microRNA.org[18], PicTar [19] and MicroCosm [20].

### Luciferase assays

A section of the 3' untranslated region (UTR) of each target containing the putative binding site(s) for miR-200c was amplified by PCR from HeLa genomic DNA using the primers listed in Table S1 in Additional file 1. Fragments were cloned into the 3' UTR of a firefly luciferase reporter vector (pMIR-REPORT, Ambion) using HindIII and SpeI. Mutations in the miR-200c binding sites were generated by PCR directed mutagenesis. Mutation primers are listed in Table S1 in Additional file 1 and introduced mutations are in bold and shown above the mRNA in each figure. 3' UTR sequences and mutations were verified by sequencing. Hec50 cells (15,000 per well) plated in a 96-well plate were mock transfected, transfected with negative control, 50 nM miR-200c, 50 nM miR-200c antagomiR (Dharmacon, Lafayette, CO, USA)) alone (α200c) or in conjunction with miR-200c (α200c + 200c). After 24 hrs, the firefly reporter plasmid (196 ng) and a *Renilla *luciferase normalization plasmid pRL-SV40 (4 ng) were introduced using Lipofectamine 2000. Cells were harvested 48 hrs later for analysis using the Dual Luciferase Reporter assay system (Promega, Madison, WI, USA)).

### Real-time reverse transcription-PCR

RNA was harvested from cells using Trizol (Invitrogen) and treated with DNase 1 (Invitrogen) for 15 minutes at room temperature. RNA was reverse transcribed into cDNA in a reaction containing reaction buffer, 10 mM DTT, 1 mM dNTPs, RNase inhibitor (Applied Biosystems), 250 ng random hexamers, and 200 units of MuLV-RT (Applied Biosystems). For normalization, real-time reverse transcription-PCR (RT-PCR) was performed on the cDNA using eukaryotic 18S rRNA endogenous control primers and FAM-MGB probe (Applied Biosystems). TaqMan MicroRNA Reverse Transcription kit was used to generate cDNA for real-time RT-PCR reaction in conjunction with a miR-200c specific primer and probe (Applied Biosystems, assay ID 002300). The reverse transcription primer for miR-200c is a hairpin primer specific to the mature miRNA and will not bind to the precursor molecules. For validation of the microarray data, SYBR Green real-time RT-PCR was performed using primers specific for each target (primers listed in Table S1 in Additional file 1). To avoid the possibility of amplification artifacts, PCR products for all SYBR Green primer pairs were verified to produce single products by agarose electrophoresis and high resolution melt curve. The relative mRNA or miRNA levels were calculated using the comparative Ct method (ΔΔCt). Briefly, the Ct (cycle threshold) values for the rRNA or actin were subtracted from Ct values of the target gene to achieve the ΔCt value. The 2^−ΔCt ^was calculated for each sample and then each of the values was divided by a control sample to achieve the relative mRNA or miRNA levels (ΔΔCt).

### Immunoblot analysis

Whole-cell protein extracts prepared in RIPA lysis buffer, equalized to 50 μg by Bradford protein assay (Bio-Rad, Hercules, CA, USA), separated by SDS-PAGE gels and transferred onto polyvinylidene difluoride (PVDF) membranes. For chemiluminecent detection, membranes were blocked in 5% milk in TBS-T and probed overnight at 4°C with primary antibodies. Primary antibodies used were ZEB1 (rabbit polyclonal from Dr. Doug Darling, University of Louisville, Louisville, KY, USA; 1:1,500 dilution), E-cadherin (clone NCH-38 from DAKO, Carpinteria, CA, USA; 1 μg/mL), fibronectin (BD Biosciences, Franklin Lakes, NJ, USA, clone 10/Fibronectin, 1:5000), moesin (Abcam, Cambridge, MA, USA, clone EP1863Y, 1:10,000), ERM (Cell Signaling, Danver, MA, USA, #3142, 1:1000), TrkB (Santa Cruz Biotechnology, Santa Cruz, CA, USA, H-181, #sc8316, 1:200) and α-tubulin (Sigma-Aldrich, St. Louis, MO, USA, clone B-5-1-2, 1:30,000). After incubation with appropriate secondary antibody, results were detected using Western Lightning Chemiluminescence Reagent Plus (Perkin-Elmer, Waltham, MA, USA). For fluorescent detection, membranes were blocked in 3% BSA (Sigma-Aldrich) in TBS-T and probed overnight at 4°C with primary antibodies. Goat anti-rabbit conjugated to Alexa Fluor 660 (Invitrogen, 1:5,000) and goat anti mouse conjugated to Alexa Fluor 660 (Invitrogen, 1:5,000) were used as appropriate and signal was detected by Odyssey (LI-COR, Lincoln, NE, USA).

### Wound healing assay

Cells were transfected with miR-200c and controls as before and 24 hrs later transfected with vectors. Cells were then plated in six-well plates, allowed to adhere and grow to confluency. Cells were then treated for two hours with 10 μg/mL mitomycin C (Fisher Scientific, Pittsburgh, PA, USA). Wounds were made using a p20 pipet tip and cells were given 24 hrs (Hec50 and BT549) or 48 hrs (AN3CA) to migrate into wounds. Cells were stained with 0.05% crystal violet in 6% glutaraldehyde for one hour, rinsed repeatedly with water, mounted and imaged. For each condition five representative images were obtained for quantitation. Quantitation was performed by first thresholding the images to differentiate between cells (black) and background (white), determining the number of black pixels and the number of white pixels and then calculating the percentage of the image covered by cells.

### Anoikis assay (cell viability and cell death ELISA)

Poly-hydroxyethyl methacrylate (poly-HEMA, Sigma-Aldrich) was reconstituted in 95% ethanol to a concentration of 12 mg/mL. To prepare poly-HEMA coated plates, 0.5 mL of 12 mg/mL solution was added to each well of a 24-well plate and allowed to dry overnight in a laminar flow tissue culture hood. Cells were transfected as before. Twenty-four hours after transfection 50,000 cells were plated in triplicate in poly-HEMA coated 24-well plates using regular culture medium. For cell viability assay, at 4 and 24 hrs after addition to poly-HEMA coated plates, viable and dead cells were stained with trypan blue and counted using the ViCell cell counter (Beckman-Coulter, Brea, CA, USA). For cell death ELISA assay (Roche, San Francisco, CA, USA) cells were plated as before, but the medium was collected at 2, 4, 8, 24 and 48 hrs post plating. Each sample was pelleted, lysed and then frozen so that all samples could be read together at 405 nm and 490 nm (reference wavelength). The assay detects fragmented mono and oligonucleosomes in lysed cells by first binding histones with a biotinylated antibody which is bound to a streptavidin-coated plate. Samples are then bound by an HRP labeled anti-DNA antibody and color is developed by using an ABTS substrate.

## Results

### Restoration of miR-200c decreases non-epithelial, EMT associated genes

**Figure 1:**
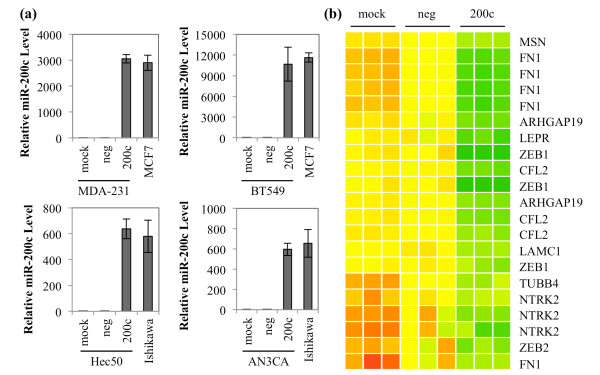
**Restoration of miR-200c decreases EMT associated genes**. **(a) **Cells were treated with transfection reagent only (mock), scrambled negative control (neg) or miR-200c mimic (200c). RNA was harvested after 72 hrs and qRT-PCR was performed for miR-200c. Samples are normalized to 18S rRNA and presented relative to mock. Columns, mean of three biological replicates, bars, standard deviation of the mean. **(b) **Heatmap of genes statistically significantly affected by restoration of miR-200c to Hec50 cells and bioinformatically predicted to be targeted by miR-200c.

We utilize breast and endometrial cancer cell lines in which we have previously characterized miR-200c levels as well as expression of classic epithelial and mesenchymal markers [11,12]. The BT549 and MDA-MB-231 cell lines are triple negative breast cancer (TNBC) cell lines, which lack expression of estrogen receptor alpha (ESR1), progesterone receptors, and HER2/neu. The TNBC lines lack E-cadherin and express the mesenchymal markers N-cadherin and vimentin and, therefore, exhibit an EMT phenotype. In contrast, MCF7 cells represent the luminal A subtype of breast cancer, which retains epithelial markers including ESR1 and E-cadherin. The Hec50 and AN3CA cell lines represent aggressive type 2 endometrial cancers that have lost epithelial markers including E-cadherin and ESR1 and gained mesenchymal markers such as N-cadherin and vimentin, indicative of EMT. In contrast, Ishikawa cells represent the less aggressive type 1 endometrial cancer, which retains epithelial markers and does not express mesenchymal markers. Transfection of miR-200c mimic into the dedifferentiated breast and endometrial cancer lines (BT549, MDA-MB-231, Hec50 and AN3CA) results in levels of mature miR-200c comparable to endogenous levels in the more well-differentiated breast and endometrial cancer lines (MCF7 and Ishikawa) (Figure 1a). These results indicate that experiments performed using this concentration of mimic result in miR-200c levels comparable to those observed in cell lines that have not undergone EMT.

By microarray expression profiling, we previously identified genes significantly altered upon restoration of miR-200c to Hec50 cells [12]. Figure 1b is a heatmap of genes known to be involved in EMT that are statistically significantly decreased at least 1.5-fold upon restoration of miR-200c and are bioinformatically predicted to be targets of miR-200c. The heatmap additionally depicts miR-200c targets identified by others such as *ZEB1 *and 2 [8,9], cofilin (*CFL1*) [9] and *WAVE3 *[21]. In total we identified 74 genes that change more than 1.5-fold and are predicted by two of four target prediction programs to be direct targets of miR-200c Figure S1 in Additional file 1. Of these genes, 68 (92%) are repressed and 6 (8%) are up-regulated when miR-200c is restored. Initial validation of several of the targets with known involvement in EMT revealed that they are down-regulated at the message level in one or more of our model cell lines Figure S2 in Additional file 1. Based on these findings, we selected *FN1*, *MSN*, *ARHGAP19*, *LEPR *and *TrkB *(*NTRK2 *on the heatmap) to experimentally confirm as direct targets of miR-200c.

### Breast and endometrial cancer cell lines that have undergone EMT and express ZEB1, also express FN1, MSN or both

**Figure 2:**
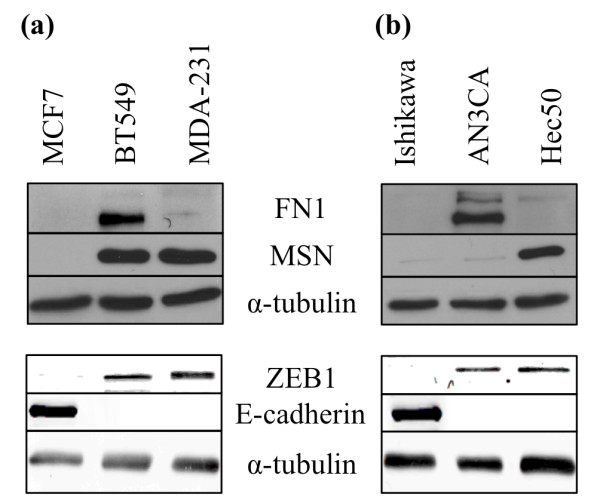
**Breast and endometrial cancer cells can express FN1 and/or MSN**. Breast **(a) **and endometrial **(b) **cancer cell lines analyzed by immunoblot for FN1, MSN, ZEB1, E-cadherin and α-tubulin expression (loading control).

Since there is substantial evidence in the literature for FN1 and MSN being involved in cancer cell migration, we assayed the breast and endometrial cancer cell lines for expression of these proteins (Figure 2). We found that neither the luminal A breast cancer cell line (MCF7) or the type 1 endometrial cancer cell line (Ishikawa) express FN1 or MSN, consistent with their pre-EMT phenotype, indicated by expression of E-cadherin and lack of ZEB1. In contrast, all of the TNBC and type 2 endometrial cancer lines express either one or both of these proteins in addition to ZEB1, supporting the hypothesis that they may play a role in migration in the absence of miR-200c.

### Moesin (*MSN*), a regulator of cortical actin-membrane binding, is directly targeted and down-regulated by miR-200c

**Figure 3:**
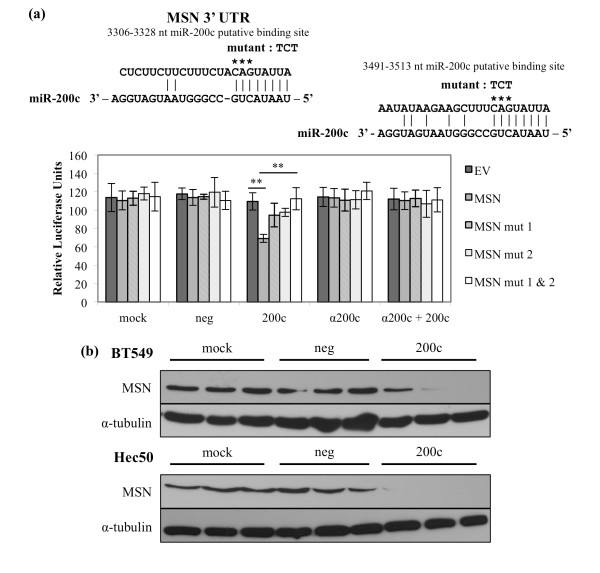
**Moesin (*MSN*), a regulator of cortical actin-membrane binding, is directly targeted and down-regulated by miR-200c**. **(a) **Regions of the 3' UTR where miR-200c is predicted to bind. Hec50 cells treated with transfection reagent only (mock), scrambled negative control (neg), miR-200c mimic (200c), miR-200c antagomiR alone (α200c) or in conjunction with miR-200c (α200c + 200c) and luciferase assay performed. Columns, mean of five replicates, bars, standard deviation of the mean. ANOVA with Tukey-Kramer post-hoc test, ** *P *< 0.01. **(b) **Immunoblot for MSN and α-tubulin (loading control) expression.

MSN connects the actin cytoskeleton and the cell membrane [22] and is strongly up-regulated in cancers with a poor prognosis, including metastatic breast cancer [23], where it contributes to migratory and invasive capacity [24-26]. The 3' UTR of *MSN *contains two putative miR-200c binding sites (Figure 3a) and we cloned the region containing these sites downstream of luciferase. When miR-200c is restored, we observe a 37% decrease in luciferase activity only in the presence of miR-200c and not the controls. To determine the specificity of this down-regulation, we mutated the putative miR-200c binding sites and observe that luciferase activity levels return to levels observed in the absence of miR-200c; thus, miR-200c binding to these sites specifically is required for down-regulation. We also observe that mutating either binding site results in a partial increase in luciferase activity, but only when both sites are mutated is there a full restoration of luciferase activity. Therefore, both binding sites are functional and required for miR-200c to exert its full effect on the *MSN *3' UTR. When an antagomiR is used to inhibit miR-200c binding to the target sites, luciferase activity is again restored. This indicates that miR-200c specifically is responsible for targeting the *MSN *3' UTR and the consequent decrease in luciferase activity. Importantly, restoration of miR-200c decreases MSN protein levels (Figure 3b) in two cell lines that express detectable MSN protein, indicating that direct targeting of *MSN *by miR-200c exerts a measurable effect on MSN protein expression.

### Down-regulation of MSN contributes to miR-200c mediated suppression of migration

**Figure 4:**
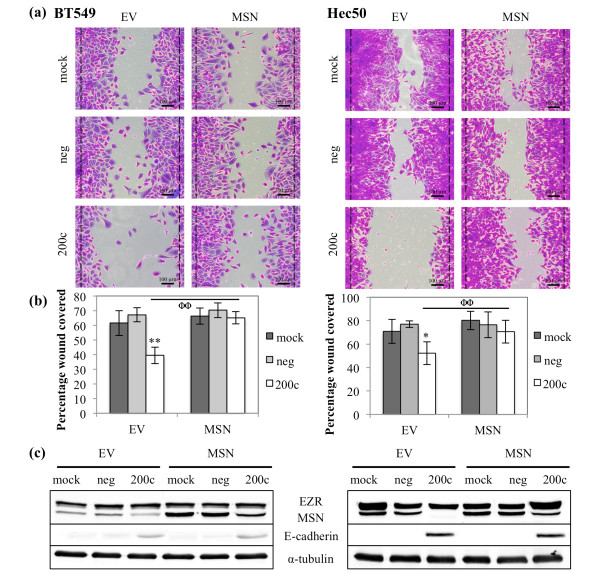
**Down-regulation of MSN contributes to miR-200c mediated suppression of migration**. Cells were transfected with empty vector (EV) or MSN and 24 hrs later with miRNA constructs. BT549 (left) and Hec50 (right) cells were treated with mitomycin C and given 24 hrs to migrate. **(a) **Brightfield images of crystal violet stained cells, dashed black lines indicate edges of the wound immediately after wounding. Scale bars are 100 μm. **(b) **Quantitation of migratory ability of cells. Columns, mean of five replicates, bars, standard deviation of the mean. ANOVA, * *P *< 0.05, ** *P *< 0.01, Tukey-Kramer post-hoc test, ΦΦ *P *< 0.01. **(c) **Immunoblot for MSN, E-cadherin and α-tubulin (loading control).

Because miR-200c decreases migration, we next sought to determine the role of MSN in the ability of miR-200c to inhibit migration. Restoration of miR-200c to BT549 and Hec50 cells results in a dramatic decrease in their ability to close a wound as indicated by movement of cells past the initial boundary of the wound (black line) (Figure 4a). BT549 cells display a 41% decrease in migratory ability, while Hec50 cells display a 32% decrease (Figure 4b). The addition of a plasmid encoding MSN lacking its 3' UTR, rendering it untargetable by miR-200c, abolishes the ability of miR-200c to decrease migration (Figure 4a, b) without further increasing the migratory ability of the mock and negative control transfected cells. This indicates that miR-200c targeting of *MSN *can play a critical role in the ability of miR-200c to decrease migration in these cell lines. The levels of MSN protein achieved with the transfection are reasonable (Figure 4c) and do not interfere with the ability of miR-200c to restore E-cadherin in these cell lines.

### The extracellular matrix protein fibronectin 1 (*FN1*) is directly targeted and down-regulated by miR-200c

**Figure 5:**
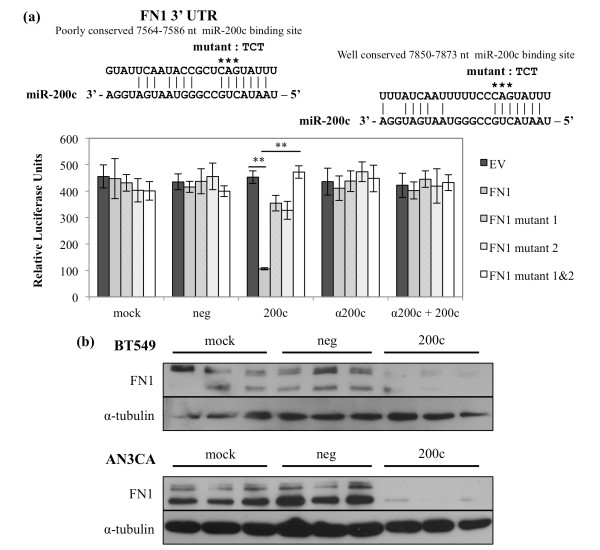
**The extracellular matrix protein fibronectin (*FN1*) is directly targeted and down-regulated by miR-200c**. **(a) **Regions of the 3' UTR where miR-200c is predicted to bind. Hec50 cells treated and luciferase assay performed. Columns, mean of five replicates, bars, standard deviation of the mean. ANOVA with Tukey-Kramer *post-hoc *test, ** *P *< 0.01. **(b) **Immunoblot for FN1 and α-tubulin (loading control) expression.

FN1 is normally expressed by fibroblasts but not epithelial cells, and is a classic marker of the EMT phenotype and tumorigenicity [27-29]. We [12] and others [8] previously observed a decrease in *FN1 *transcript upon restoration of miR-200c and we sought to determine if this is due to direct targeting. Like *MSN*, *FN1 *contains two putative miR-200c binding sites in its 3' UTR. When miR-200c is restored, we observe a 76% decrease in luciferase activity only in the presence of miR-200c and not in the controls (Figure 5a). As for *MSN*, mutated constructs show that miR-200c binding to these sites specifically is required for down-regulation and both binding sites are functional and required for miR-200c to exert its full effect on the *FN1 *3' UTR. When an antagomiR is used to inhibit miR-200c binding to the target sites, luciferase activity is again restored. This indicates that miR-200c specifically is responsible for targeting the *FN1 *3' UTR and the consequent decrease in luciferase activity. Only the AN3CA and BT549 express detectable protein levels (Figure 2) and restoration of miR-200c to these cell lines dramatically decreases FN1 protein expression (Figure 5b).

### Down-regulation of FN1 contributes to miR-200c mediated suppression of migration

**Figure 6:**
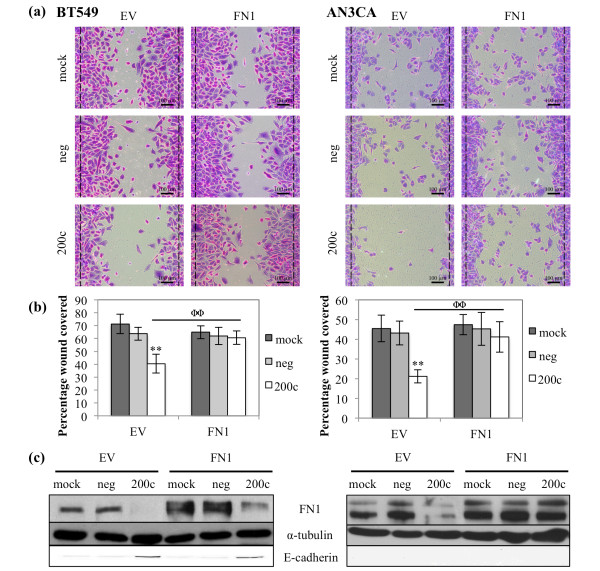
**Down-regulation of FN1 contributes to miR-200c mediated suppression of migration**. Cells were transfected with empty vector (EV) or FN1 and 24 hrs later with miRNA constructs. BT549 (left) and AN3CA (right) cells were treated with mitomycin C and given 24 or 48 hrs, respectively, to migrate. **(a) **Brightfield images of crystal violet stained cells, dashed black lines indicate edges of the wound immediately after wounding. Scale bars are 100 μm. **(b) **Quantitation of migratory ability of cells. Columns, mean of five replicates, bars, standard deviation of the mean. ANOVA, ** *P *< 0.01, Tukey-Kramer post-hoc test, ΦΦ *P *< 0.01. **(c) **Immunoblot for FN1, E-cadherin and α-tubulin (loading control).

We next sought to determine if FN1 plays a role in miR-200c control of migration. Restoration of miR-200c to BT549 and AN3CA cells again results in a dramatic decrease in migration (Figure 6a), which is abrogated by addition of an untargetable *FN1 *plasmid. The BT549 cells exhibit a 43% decrease in migratory ability, while the AN3CA cells decrease 53% (Figure 6b). Thus, down regulation of FN1 is an additional mechanism by which miR-200c suppresses migration in aggressive breast and endometrial cancer cell lines. The levels of FN1 protein achieved with the plasmid are reasonable and do not interfere with the ability of miR-200c to restore E-cadherin expression in the BT549 cell (Figure 6c). The AN3CA cells do not re-express E-cadherin following restoration of miR-200c.

### The genes encoding Rho GTPase activating protein 19 (*ARHGAP19*) and leptin receptor (*LEPR*) are directly targeted and down-regulated by miR-200c

ARHGAP19 is a GTPase activating protein that has not been well characterized, but is predicted to regulate the activity of Cdc42, RhoA and/or Rac1 [30]. The 3' UTR of *ARHGAP19 *contains one putative miR-200c binding site. We demonstrate that restoration of miR-200c causes an 80% reduction in luciferase activity only in the presence of miR-200c and not in the controls (Figure S3 in Additional file 1). LEPR and its ligand leptin are involved in the migration/invasion of trophoblasts [31] and the expression of leptin by mammary epithelial cells has been linked to tumorigenicity [32-34]. We demonstrate that restoration of miR-200c causes a 36% reduction in luciferase activity when the 3' UTR of *LEPR *is placed downstream of luciferase (Figure S4 in Additional file 1).

### The anoikis suppressing neurotrophic receptor tyrosine kinase 2 (*NTRK2 *or *TrkB*) is directly targeted and down-regulated by miR-200c

**Figure 7:**
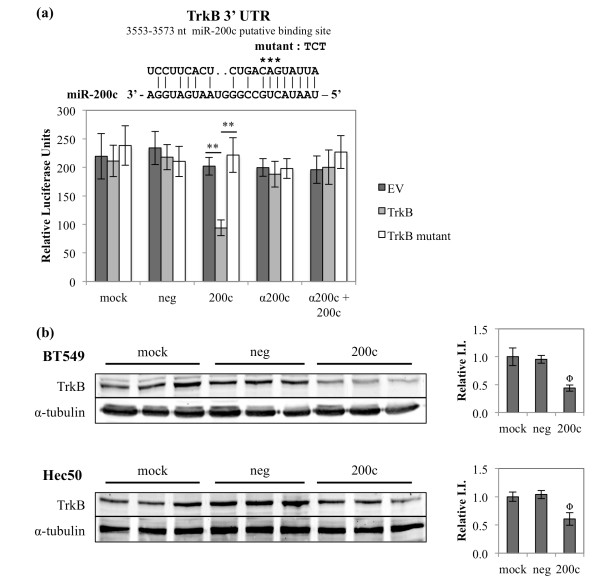
**The anoikis suppressing neurotrophic receptor tyrosine kinase 2 (*NTRK2 *or *TrkB*) is directly targeted and down-regulated by miR-200c**. **(a) **The region of the 3' UTR where miR-200c is predicted to bind. Hec50 cells treated and luciferase assay performed. Columns, mean of five replicates, bars, standard deviation of the mean. ANOVA with Tukey-Kramer post-hoc test, ** *P *< 0.01. **(b) **(Right) Immunoblot for TrkB and α-tubulin (loading control) expression. (Left) Quantitation of TrkB integrated intensity (I.I.), normalized to α-tubulin and presented relative to mock. ANOVA, Φ *P *< 0.05.

TrkB expression leads to anoikis resistance in several types of cancer, including breast [35-38], and this led us to investigate the regulation of this cell surface receptor by miR-200c. We demonstrate that *TrkB *is a direct target of miR-200c, showing a 55% reduction in luciferase activity (Figure 7a). Luciferase activity is restored following either mutation of the binding site or addition of an antagomiR, indicating that miR-200c binds to the 3' UTR of *TrkB *to downregulate it. Additionally, restoration of miR-200c significantly decreases endogenous TrkB protein in the BT549 and Hec50 cells (Figure 7b).

### miR-200c suppresses anoikis resistance

**Figure 8:**
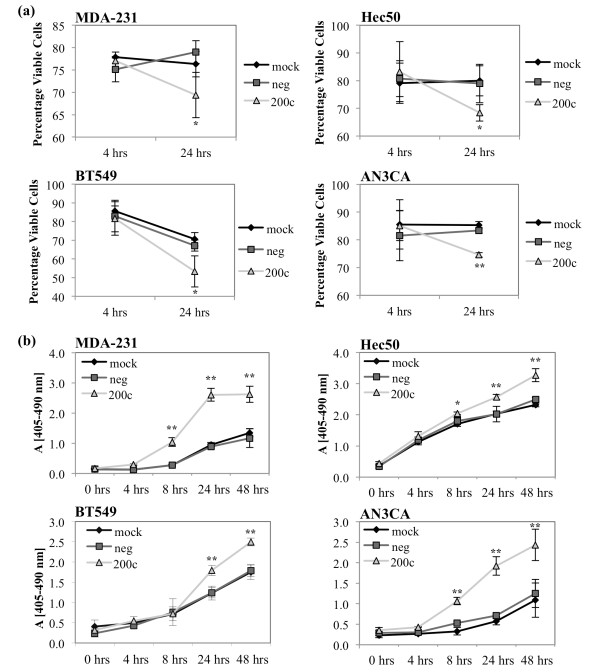
**miR-200c increases sensitivity to anoikis**. Breast (left) and endometrial (right) cancer cells were transfected with miRNA constructs and plated on poly-HEMA coated plates. Cells were collected for viability analysis by trypan blue exclusion **(a) **or apoptosis analysis by cell death ELISA **(b)**. Columns, mean of three biological replicates, bars, standard deviation of the mean. ANOVA, * *P *< 0.05, ** *P *< 0.01.

Given the known role of TrkB in anoikis resistance, we investigated the effect of miR-200c on anoikis by performing cell viability assays and cell death ELISAs. In these assays the cells are plated on poly-HEMA coated plates, which prevents them from adhering. The cells are forced to float in suspension for the times indicated before being harvested for analysis. Cell viability was determined by trypan blue exclusion and shows that restoration of miR-200c significantly decreases viability as quickly as 24 hrs in suspension (Figure 8a). In the cell death ELISAs, restoration of miR-200c results in an increase in fragmented nucleosomes, indicating an increase in apoptosis in these samples (Figure 8b). Thus, restoration of miR-200c decreases anoikis resistance as indicated by a decrease in the viability of suspended cells and concurrent increase in apoptosis.

### Down-regulation of TrkB contributes to miR-200c mediated suppression of anoikis resistance

**Figure 9:**
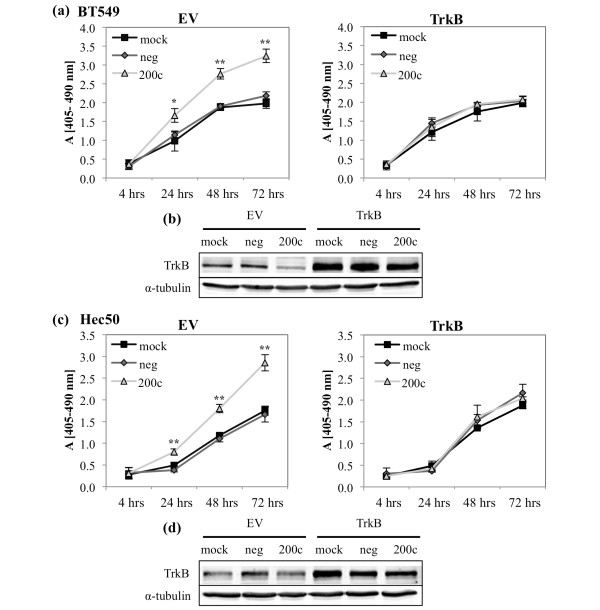
**Down-regulation of TrkB contributes to miR-200c mediated suppression of anoikis resistance**. Cells were transfected with empty vector (EV) (left) or TrkB (right) and 24 hrs with miRNA constructs. Twenty-four hours later cells were plated on poly-HEMA coated plates and cell death ELISA performed at time points indicated **(a) **and **(c)**. Columns, mean of three biological replicates, bars, standard deviation of the mean. ANOVA, * *P *< 0.05, ** *P *< 0.01. **(b) **and **(d) **Immunoblot for TrkB and α-tubulin (loading control).

To determine if targeting of *TrkB *is responsible for the ability of miR-200c to restore sensitivity to anoikis, we used a plasmid encoding *TrkB *lacking the 3' UTR, rendering it untargetable by miR-200c. Restoration of miR-200c enhances sensitivity to anoikis (Figures 8 and 9), but this phenotype is completely reversed in the presence of exogenous, untargetable *TrkB *(Figure 9a, c). However, it is important to note that the addition of exogenous TrkB does not decrease the amount of cell death in mock or negative control transfected cells. This indicates that miR-200c targeting of *TrkB *plays a critical role in the ability of miR-200c to reverse anoikis resistance.

## Discussion

Progression and metastasis of carcinomas is a multistep process. EMT is thought to aid cancer cells as they invade through basement membrane and stroma, intravasate into blood or lymph vessels, and may also facilitate anoikis resistance, allowing tumor cells to survive the journey to the metastatic site. We sought to identify additional direct targets of miR-200c that mediate its potent effects.

Three of the new direct targets of miR-200c that we identify, *MSN*, *FN1*, and *ARHGAP19*, have been implicated in migration and invasion. MSN localizes to the trailing edge of invasive melanoma cells and disruption of this localization leads to decreased metastasis [25]. MSN expression correlates with poor prognosis in oral squamous cell carcinoma [24] and basal breast cancer [23], a subtype with high risk of metastasis and recurrence. FN1 functions in cell migration through integrin binding [39] and can activate focal adhesion kinase (FAK) leading to increased motility and invasion of carcinoma cells [27,28]. ARHGAP19 is a member of a family of GTPase activating proteins, and other family members, 8, 9, 12 and 15, are expressed in several types of cancer and activate Cdc42, Rac1 or RhoA [40-43], small GTPases required for migration. We demonstrate that *FN1 *and *MSN *are, at least in some cell lines, critical targets sufficient to mediate miR-200c's ability to inhibit migration in an *in vitro *wound healing assay. In some cell lines both MSN and FN1 are expressed, and in those cells both MSN and FN1 may contribute to migratory potential, but they are both repressed when miR-200c is restored. In other TNBC cells and type 2 endometrial cancer cells, either MSN or FN1 are expressed but not both. It is possible that even though miR-200c is absent, additional miRNA(s) that target these genes may be retained in some cells, or alternatively, factors that induce these genes at the promoter may be differentially expressed. In some cases ARHGAP19 may additionally contribute to migratory capacity; however, at present there is no antibody available to detect this protein. Loss of miR-200c could permit any of these genes, typically expressed in the more motile mesenchymal or neuronal cell types, to be inappropriately translated and expressed in epithelial cells. Expression of proteins such as MSN that actively contribute to cell motility by promoting front-rear polarity, combined with the loss of E-cadherin (which would decrease cell-cell attachments and reduce apical-basal polarity), may significantly contribute to the invasive capacity of carcinomas.

We demonstrate that restoration of miR-200c leads to a dramatic increase in sensitivity to anoikis (over a 100% increase in anoikis in some cell lines) and identify *TrkB *as a novel direct target of miR-200c. TrkB is a tyrosine kinase cell surface receptor typically expressed on neurons, which can be inappropriately expressed in carcinomas [44]. In breast and ovarian cancer cell lines TrkB induces anoikis resistance [31,33] and can induce EMT through activation of Twist [41]. We previously demonstrated that miR-200c does not affect apoptosis when endometrial cancer cells are attached to plastic, although it does enhance apoptosis induced by taxanes [11,12]. Thus, we conclude that miR-200c specifically enhances anoikis sensitivity, suggesting that restoration of miR-200c could limit the ability of breast and endometrial cancer cells to survive in the bloodstream.

Interestingly, all of the new miR-200c direct targets that we identify in this study (as well as other previously identified targets such as ZEB1/2 and TUBB3) contribute to the designation of this miRNA as a "guardian of the epithelial phenotype" because they are genes typically expressed in cells of mesenchymal or neuronal origin, but not in normal, well-differentiated epithelial cells.

Not all of the target genes that we identify change at the message level upon restoration of miR-200c. For example, although miR-200c directly targets *ARHGAP19 *(Figure S3 in Additional file 1), the message is down-regulated by addition of miR-200c in only 3 of 4 cell lines (Figure S2 in Additional file 1). There are several possible explanations for interference between a miRNA and its mRNA target in some cell lines. The miR-200c target site may be mutated or absent due to a shortening of the 3' UTR [46-49] or there may be RNA binding proteins present in particular cell lines that prevent miR-200c from binding [50]. Importantly, for all of the targets that we follow up on in this study (MSN, FN1 and TrkB), protein levels are affected by miR-200c, indicating that it does have an affect on translation of these genes, regardless of whether it also affects degradation of the message.

## Conclusions

In summary, miR-200c inhibits migration and invasion [9-13], stemness [51,52], and chemoresistance [11,12] and we now identify a completely novel role for miR-200c - the ability to reverse anoikis resistance, an important additional step in the metastatic cascade. We identify new targets of miR-200c, which together with previously identified targets, comprise a program of genes normally restricted to cells of mesenchymal or neuronal origin. We specifically pinpoint *MSN *and *FN1 *as well as *TrkB *as targets that can respectively mediate the ability of miR-200c to inhibit cell motility and anoikis resistance.

Members of the miR-200 family are down-regulated in breast cancer stem cells and normal mammary gland stem cells [51]. Polycomb complexes facilitate stem cell self-renewal and pluripotency, and both Bmi1, a component of the PRC1 polycomb complex, and Suz12, a component of the PRC2 polycomb complex, have been identified as targets of miR-200 family members [51-53]. It is interesting to speculate as to whether expression of TrkB is involved in the ability of cancer stem cells to resist anoikis.

If feasible, effective *in vivo *delivery of miR-200c could potentially inhibit multiple steps in tumor progression, including tumor formation, cell motility/invasiveness, anoikis resistance and chemoresistance, by virtue of simultaneously repressing multiple, yet specific, targets expressed in carcinoma cells exhibiting an EMT phenotype. Although one *in vivo *study demonstrated that introduction of miR-200c reduced the ability of primary human breast cancer stem cells to form tumors in immune compromised mice [51], further *in vivo *studies will be necessary to specifically isolate the effects of miR-200 on other steps in the metastatic cascade, such as its potential to reverse anoikis resistance.

## Abbreviations

DMEM: Dulbecco's modified eagle's medium; EMT: epithelial to mesenchymal transition; ESR1: estrogen receptor alpha; FAK: focal adhesion kinase; FBS: fetal bovine serum; FN1: fibronectin 1; LEPR: leptin receptor; MSN: moesin; NEAA: non-essential amino acids; poly-HEMA: poly-hydroxyethyl methacrylate; TNBC: triple negative breast cancer; UTR: untranslated region.

## Competing interests

The authors declare that they have no competing interests.

## Authors' contributions

ENH performed experimental studies. DRC performed array profiling studies and generated the heatmap in Figure 1. All authors contributed intellectual input towards the design, implementation, and interpretation of results. ENH and JRK drafted the manuscript and all authors read and approved the final manuscript.

## Supplementary Material

Additional file 1 **Additional experimental data and the sequences of primers used in cloning and qRT-PCR**. Click here for file
