# Supplementary material for: Targets of miR-200c mediate suppression of cell motility and anoikis resistance
Source: Breast Cancer Res. 2011 Apr 18;13(2):R45. doi: 10.1186/bcr2867 (PMC3219208; doi:10.1186/bcr2867)
Supplement: Additional file 1 — Additional experimental data and the sequences of primers used in cloning and qRT-PCR. [file bcr2867-S1.PDF]

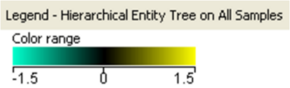

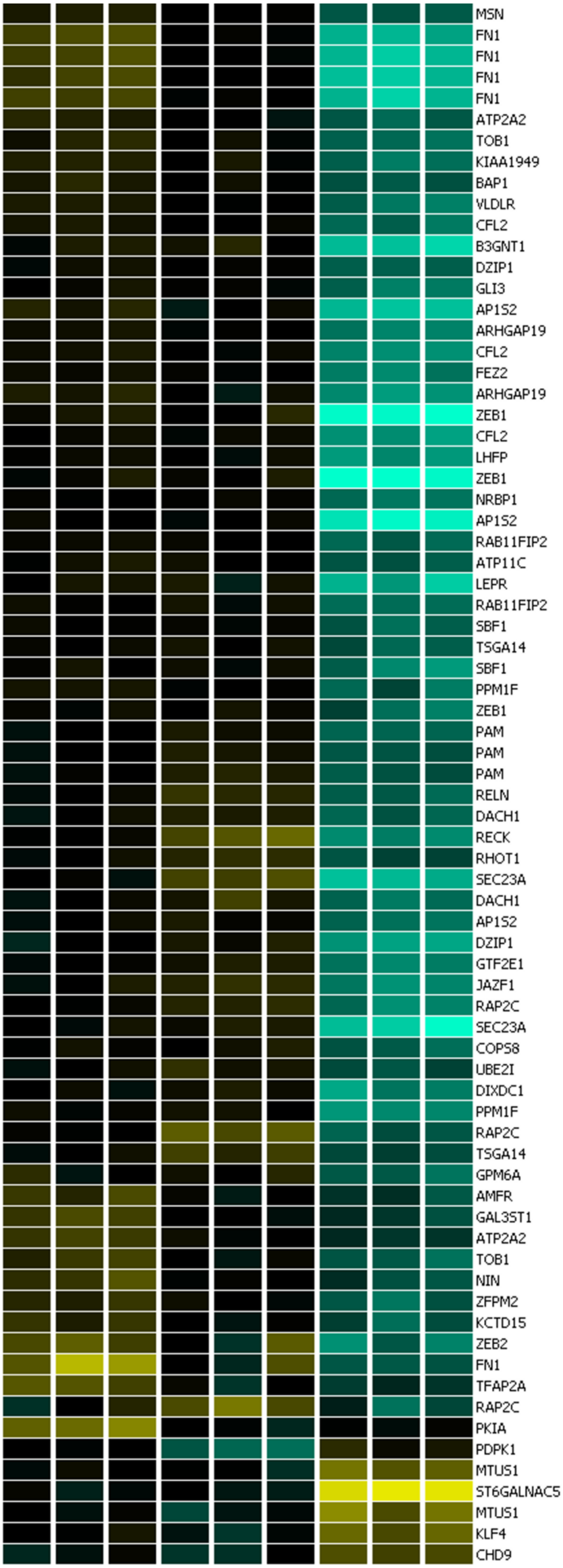

Figure S1: Genes differentially regulated and bioinformatically predicted to be directly targeted by miR-200c. Heatmap shows genes that are statistically significantly > 1.5 fold down (blue) or upregulated (yellow) following restoration of miR-200c. These genes are also bioinformatically predicted to be directly targeted by miR-200c as predicted by two or more of the following programs: TargetScan, microRNA.org, PicTar or miRbase.

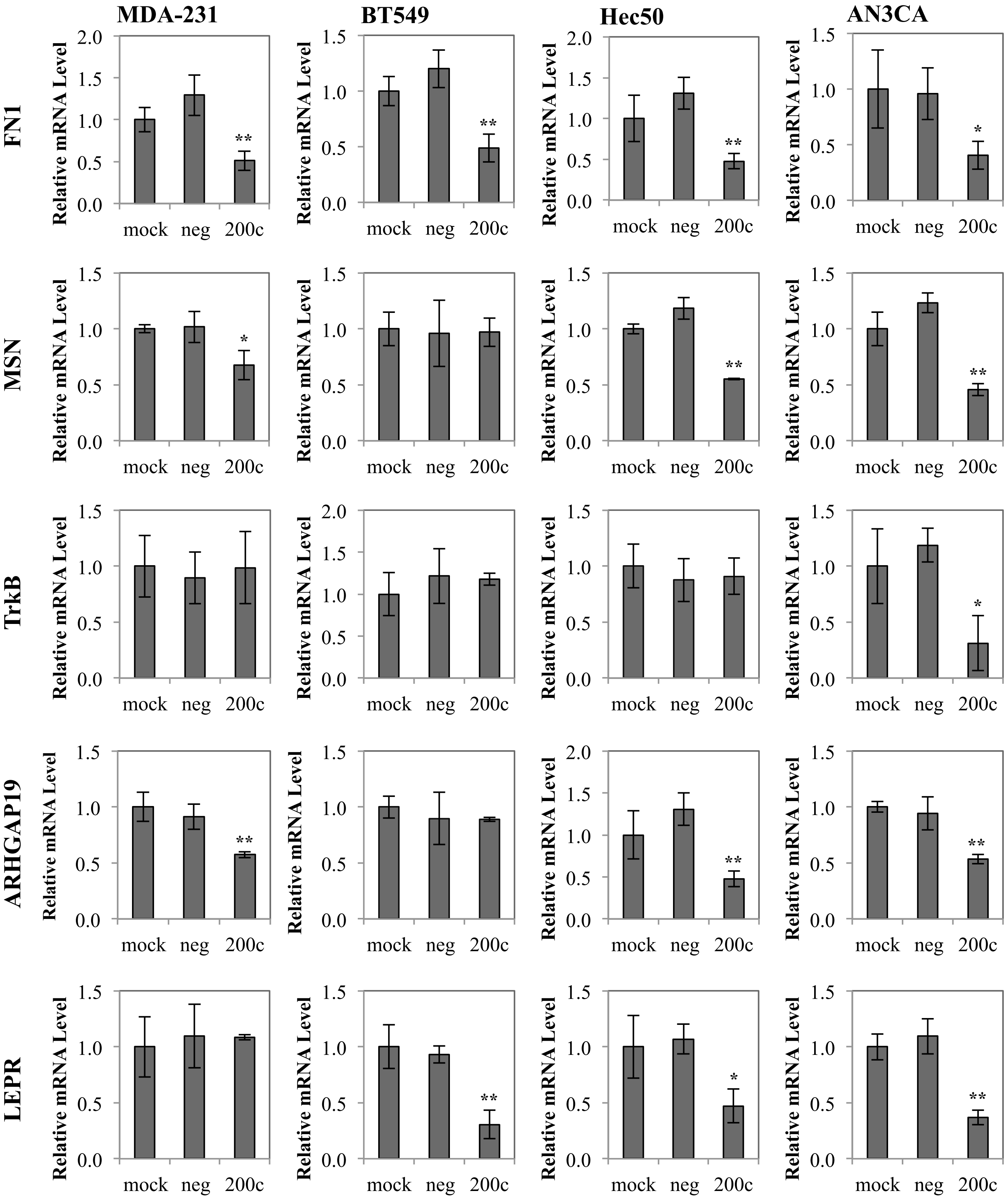

Figure S2: Validation of microarray results for genes of interest. Breast (MDA-231 and BT549) and endometrial (Hec50 and AN3CA) cancer cells were transfected with miRNA constructs and 72 hrs later harvested for RNA and qRT-PCR was performed for each gene. Results are normalized to actin levels and presented relative to mock. ANOVA, * $p < 0.05$, ** $p < 0.01$.

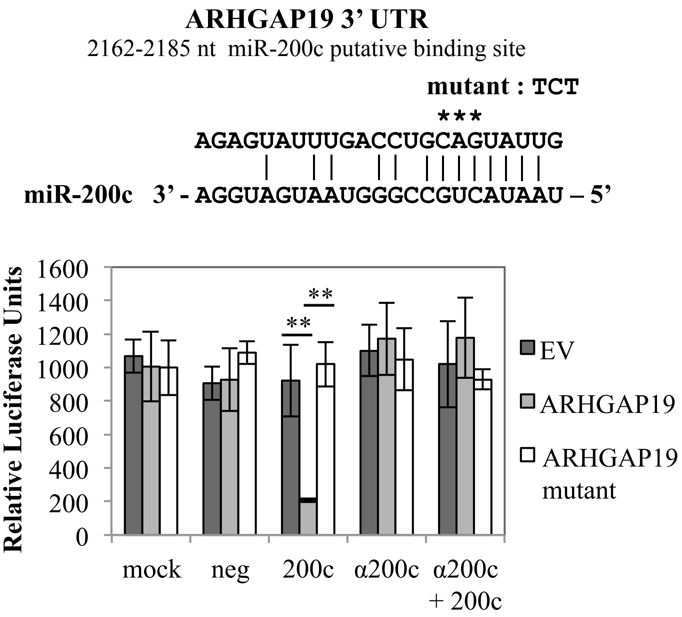

Figure S3. RhoGTPase activating protein 19, ARHGAP19, is directly targeted and down-regulated by miR-200c. Regions of the 3' UTR where miR-200c is predicted to bind. Hec50 cells treated with transfection reagent only (mock), scrambled negative control (neg), miR-200c mimic (200c), miR-200c antagomiR alone (α200c) or in conjunction with miR-200c (α200c + 200c) and luciferase assay performed. *Columns*, mean of five replicates, *bars*, standard deviation of the mean. ANOVA with Tukey-Kramer post-hoc test, ** $p < 0.01$.

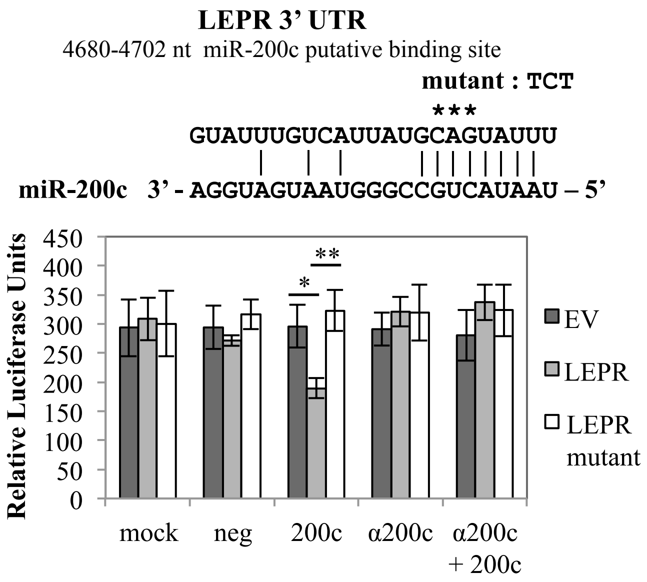

Figure S4. The leptin receptor, LEPR, is directly targeted and down-regulated by miR-200c. Regions of the 3’ UTR where miR-200c is predicted to bind. Hec50 cells treated with transfection reagent only (mock), scrambled negative control (neg), miR-200c mimic (200c), miR-200c antagomiR alone (α200c) or in conjunction with miR-200c (α200c + 200c) and luciferase assay performed. *Columns*, mean of five replicates, *bars*, standard deviation of the mean. ANOVA with Tukey-Kramer post-hoc test, ** $p < 0.01$.

LEPR, a 153 bp section was cloned.
LEPR F 5' – CCACTAGTCAGGCATAGGAACA – 3'
LEPR R 5' – CTCAAGCTTTGCCAAGCGCA – 3'
LEPR mut F 5' – TATGCA**TCT**TTTAATACCTACATAAG – 3'
LEPR mut R 5' – TTAAAA**AGA**TGCATAATGACAAATACT – 3'

ARHGAP19, a 321 bp section was cloned.
ARHGAP19 F 5' – CCACTAGTGGCTGCATTCCT – 3'
ARHGAP19 R 5' – CTCAAGCTTCACATCACTGA – 3'
ARHGAP19 mut F 5' – ACCTGCA**TCT**TTGAAAAAGGAGAATTCA – 3'
ARHGAP19 mut R 5' – TTTCAA**AGA**TGCAGGTCAAATACTCTG – 3'

TrkB, a 166 bp fragment was cloned.
TrkB F 5' – CCACTAGTAGACCGATCCTT – 3'
TrkB R 5' – CTCAAGCTTAGTACACACTGC – 3'
TrkB mut F' – CTGACA**TCT**TAACATCAAAGACTCCGA – 3'
TrkB mut R 5' – TGTTAA**AGA**TGTCAGAGTGAAGGAGAG – 3'

FN1, a 510 bp fragment was cloned.
FN1 F 5' – CCACTAGTCAGCTTCAGCTCA – 3'
FN1 R 5' – CTCAAGCTTGGCACATACAGT – 3'
FN1 mut 1 F 5' – ACCGCTCA**TCT**TTTTAAATGAAGTTTT – 3'
FN1 mut 1 R 5' – TTAAAA**AGA**TGAGCGGTATTGAATACT – 3'
FN1 mut 2 F 5' – TTCCCA**TCT**TTTTTATACGGAAAAAAT – 3'
FN1 mut 2 R 5' – TAAAAA**AGA**TGGGAAAAATTGATAAAT – 3'

MSN, a 390 bp fragment was cloned
MSN F 5' – CCACTAGTCAGCTTCAGCTCA – 3'
MSN R 5' – CTCAAGCTTGGCACATACAGT – 3'
MSN mut 1 F 5' – TCTACA**TCT**TTATGTACTCTACTGATA – 3'
MSN mut 1 R 5' – ACATAA**AGA**TGTAGAAAGAAGAAGAGC – 3'
MSN mut 2 F 5' – CTTTCA**TCT**TTAGTGATGTCATCTGTC – 3'
MSN mut 1 R 5' – CTCTAA**AGA**TGAAAGCTTCTTATATTA – 3'

ARHGAP19 F 5' – TGTGGCTTGTCACCAATGTT – 3'
ARHGAP19 R 5' – CACAGGGTGAGAAGGGTTGT – 3'

LEPR F 5' – ATTGGAGCAATCCAGCCTAC – 3'
LEPR R 5' – CAGGGGCTTCCAAAGTAAAG – 3'

FN1 F 5' – TCGAGGAGGAAATTCCAATG – 3'
FN1 R 5' – CTCTTCATGACGCTTGTGGA – 3'

MSN F 5' – ACAGTCGCCCCGACGCTAGT – 3'
MSN R 5' – TTGGGCATGGTGGCGGCAAA – 3'

TrkB F 5' – CCTGCTGGGTAGTGGCTGCG – 3'
TrkB R 5' – CATGGCATCCGTGTGGCCGT – 3'

Table S1: Primers used for PCR, mutagenesis and qRT-PCR.
